# A Bibliometric Analysis of the Hotspots Concerning Stem Cell Extracellular Vesicles for Diabetes in the Last 5 Years

**DOI:** 10.3389/fpubh.2022.868440

**Published:** 2022-06-02

**Authors:** Haiyuan Qiu, Rong Guo, Yuanwen Zhang, Jianghui Ying, Yazhou Yan, Jiachao Xiong

**Affiliations:** ^1^Department of Internal Medicine, Ningbo Psychiatric Hospital, Ningbo, China; ^2^Department of Plastic Surgery, Shanghai East Hospital, Tongji University School of Medicine, Shanghai, China; ^3^Department of Burns and Plastic Surgery, No. 926 Hospital, Joint Logistics Support Force of PLA, Kaiyuan, China; ^4^Department of Neurosurgery, 971 Hospital of PLA, Qingdao, China

**Keywords:** diabetes mellitus, stem cells, extracellular vesicles, bibliometric analysis, hotspot

## Abstract

**Background:**

Diabetes mellitus (DM) is a metabolic disease that endangers human health, and its prevalence is exploding and younger. Stem cell-derived extracellular vesicles (SC-EVs) have a repair function similar to SCs and no risk of tumor formation, which have been widely used in the repair of DM and its complications. We aim to map the hot trends of SC-EVs for the treatment of DM and providing directions for future research.

**Methods:**

We screened all relevant publications on SC-EVs for DM from the Web of Science (Wos) during 2017–2021, and research trends in this field were analyzed by VOSviewer and CiteSpace.

**Results:**

A total of 255 articles related to SC-EVs for DM were screened out according to the search strategy. China (122 publications and 2,759 citations) was the most productive country, followed by the USA (50 publications and 1,167 citations) and Italy (16 publications and 366 citations). The top five institutions with the most publications were located in Italy and China, with Turin University being the most productive. The journals Stem Cell Research and Therapy and International Journal of Molecular Sciences published most of the studies on SC-EVs for DM. ASHOK KUMAR published the majority of articles in this field, while QING LI was the most cited. Cluster analysis indicated that the current research trend is more focused on the repair mechanism and clinical translation of exosomes and their related preparations in promoting DM and its complications.

**Conclusion:**

In this study, a comprehensive summary and analysis of the global research trends of SC-EVs used in DM and its complications was performed. In the past 5 years, relevant high-quality publications in this field have increased significantly, and SC-EVs have a good prospect for development in the treatment of DM and its complications.

## Introduction

Diabetes mellitus (DM) is a metabolic disease whose main manifestation is chronic hyperglycemia, combined with defective insulin secretion or action, and is one of the major diseases endangering human health, and its prevalence is exploding and younger (1). According to a recent report by the International Diabetes Federation (http://www.diabetesatlas.org/), in 2021, ~537 million adults (20–79 years old) are living with DM worldwide, and this number is predicted to rise to 643 million in 2030 and 783 million in 2045. Notably, DM causes 6.7 million deaths in 2021 and is responsible for at least USD 966 billion in health expenditure. Patients with diabetes in a continuous state of elevated glucose can lead to the development of various complications, such as diabetic wound, nephropathy, and retinopathy (2). Unfortunately, DM cannot be cured at present, and the primary way to strictly control hyperglycemia is to improve the patient's lifestyle habits, such as diet and exercise, combined with medication to prevent and delay the development of related complications and improve the quality of life. However, this requires a high degree of patient compliance, which is difficult to achieve for many patients. At the same time, existing treatments are slightly inadequate for patients who have developed diabetic complications. Therefore, there is an urgent need for new approaches to treat DM and its related complications.

Stem cells (SCs) are a class of pluripotent progenitor cells with multidirectional differentiation potential, which have been widely used in tissue regeneration engineering due to their abundant source, easy extraction and expansion, and remarkable tissue repair effects confirmed by numerous studies (3). SCs have the role of delaying the progression of DM and promoting the repair of diabetic complications through immunomodulation, vascularization, and modulation of graft-vs.-host response to recover the insulin sensitivity of peripheral tissues, improve the peri-islet microenvironment, and upgrade islet β-cell regeneration (4–6). However, the challenges of SC storage and transport and the risk of pro-tumor formation have not yet been overcome, which greatly limit the clinical translation of SC therapies (7–9).

Extracellular vesicles (EVs) are lipid bilayer vesicles secreted by most cells and are classified into exosomes (30–150 nm), microsomes (100–1,000 nm), and apoptotic vesicles (1–5 μm) based on their diameters. The secreted EVs, which contain various substances, such as lipids, proteins, and noncoding RNAs, act on adjacent target cells *via* autocrine or paracrine secretion, or act on specific and distant target cells through humoral transport, and then act directly on target cells through membrane fusion or endocytosis to participate in complex and delicate intercellular communication. Previous studies have found that SC-derived EVs (SC-EVs) have a repair function similar to SCs and no risk of tumor formation (10, 11). Notably, numerous studies have shown that SC-EVs (especially exosomes) exert anti-inflammatory, anti-apoptotic, and pro-vascularization mechanisms through their abundant growth factors and therapeutic noncoding RNAs to promote the repair of organs damaged by DM and diabetic complications (2, 12).

Literature is a carrier that can record scientific progress. Bibliometrics uses quantitative methods such as mathematics and statistics to study the internal connections and distribution patterns among the literature to discover the current state of research, research hotspots, and future trends in a certain field (13). As research on the repair potential of SC-EVs in DM and its related complications has made tremendous progress, the number of related publications has increased dramatically, but relevant bibliometric studies have not been reported. In this study, we analyzed the publications on SC-EVs for DM and its related complications in the Web of Science (WoS) database during 2017–2021, in which the number of publications, institutions, and keywords were statistically analyzed with the aim of mapping hot trends in SC-EVs for the treatment of DM and providing directions for future research.

## Materials and Methods

### Data Collection Strategy

**Figure 1:**
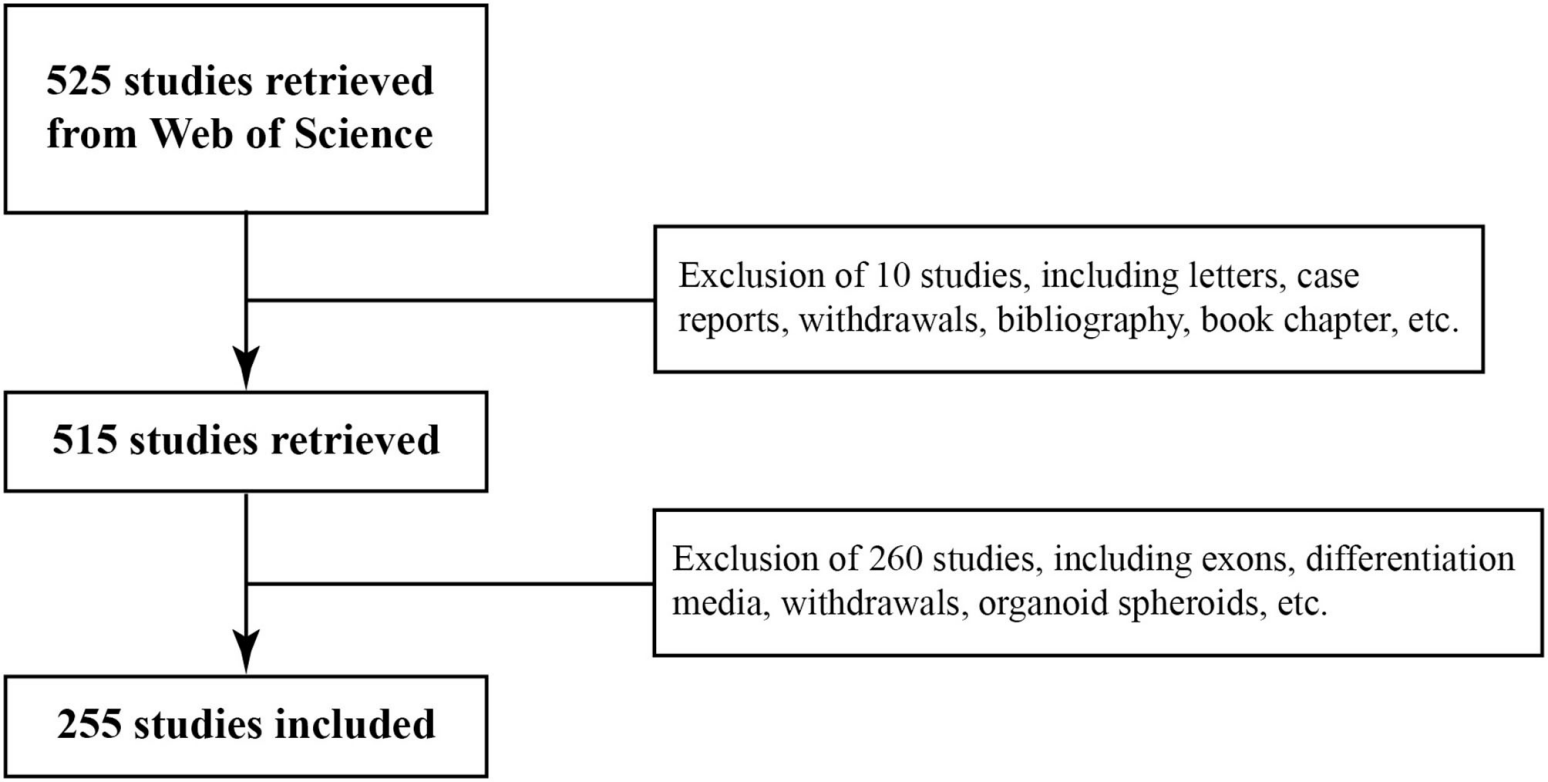
Flow chart of the screening process for research on stem cell-derived extracellular vesicles (SC-EVs) for diabetes.

Web of Science is an authoritative academic database that has been widely used by researchers worldwide. In this study, the WoS core collection was selected as the retrieval data source of SC-EVs for DM from 2017 to 2021. The search strategy and screening process were as follows (Figure 1; Supplementary Figure 1): TS = (stem cell^*^ OR SC) AND TS = (diabet^*^ OR diabetes mellitus OR DM) AND TS = (extracellular vesicle^*^ OR EV OR exo^*^) AND Language = (English) AND Publication Date = (2017-01-01 to 2021-12-31). All documents that included the above search strategy were reviewed, but letters, case reports, withdrawals, bibliography, etc. were excluded. Publicly available data sets were analyzed in this study, and ethics statement was not required. All searches were conducted on 14 January 2022 to avoid bias related to database updates.

### Data Collection

Two authors (HQ and RG) independently retrieved data and excluded studies irrelevant to the collection strategy. All data, including title, keywords, authors, institutions, etc., were extracted from the WoS and were eventually included in this study. A bibliometric analysis was performed using Microsoft Excel 2021, CiteSpace V, and VOSviewer.

### The Bibliometric Analysis Method

Based on the data extracted from the WoS, we first analyzed the publication and citation trends of SC-EVs for diabetes and visualized them using Excel. Then, bibliometric analyses, including country and institution bibliographic coupling analysis, reference co-citation analysis, and keyword co-occurrence, were performed and visualized using CiteSpace V and VOSviewer. The newest edition of the Journal Citation Report (JCR) was used to obtain the latest impact factors (IF). The scimago journal and country rank (https://www.scimagojr.com/) and eigenfactor (http://www.eigenfactor.org/index.php) websites were used to obtain the H-index and eigen factor score, respectively.

## Results

### Publishing Trends and Global Contributions

**Figure 2:**
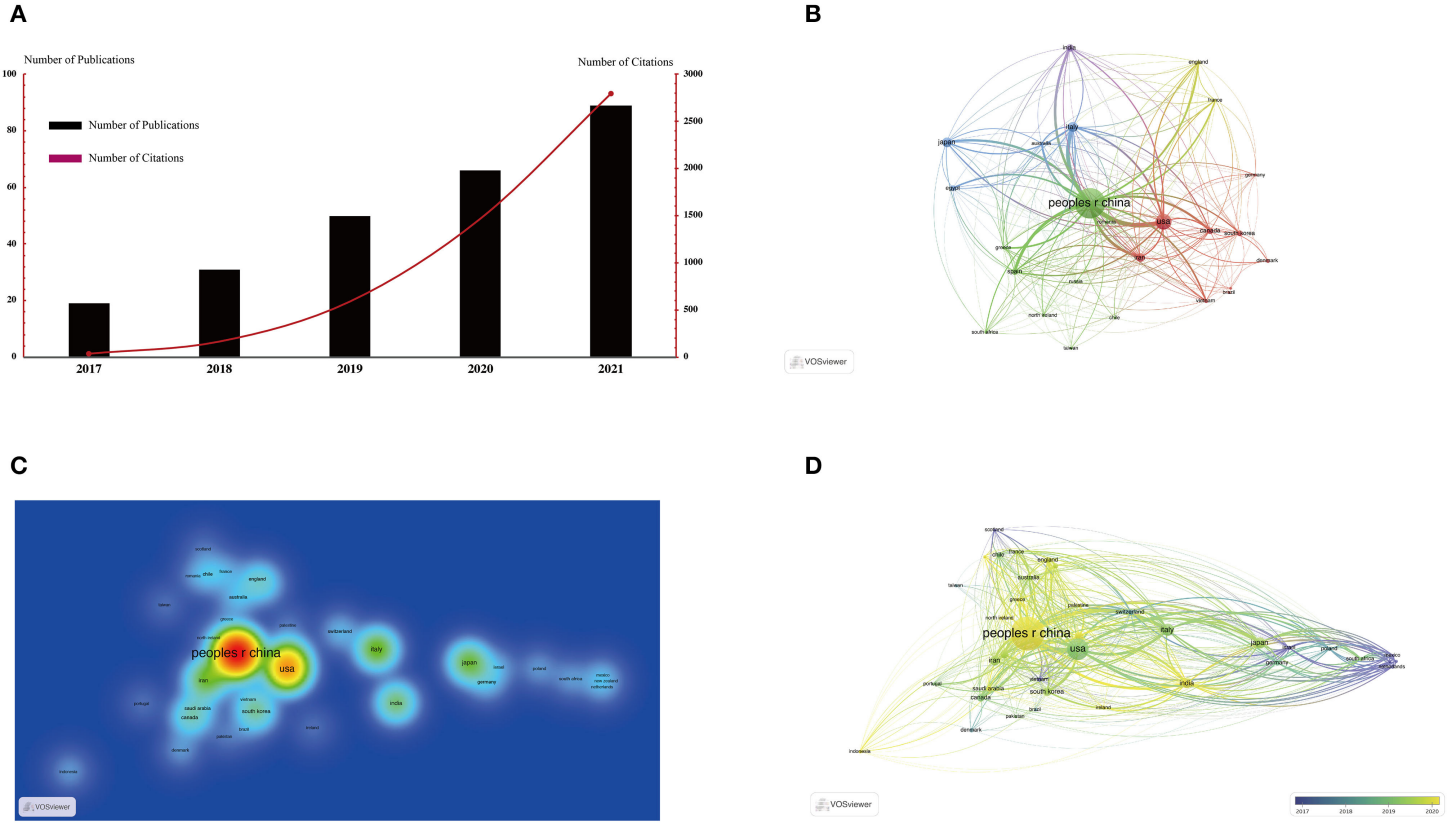
Articles related to SC-EVs for diabetes published worldwide. **(A)** Annual publications and citations worldwide of SC-EVs for diabetes. **(B)** Network visualization of the country distribution of articles based on VOSviewer. The size of the circles signifies the number of publications, and the width of the connecting line between the two circles signifies the intensity of collaboration. **(C)** Density map of the country distribution of articles based on VOSviewer. **(D)** Chronological order of countries based on VOSviewer.

A total of 255 articles were related to SC-EVs for DM according to the search strategy and were included in the final bibliometric analysis. Based on the number of annual publications (Figure 2A), the overall trend of publication research has significantly increased year by year, especially 2018–2019 (31–50 publications per year) and 2020–2021 (66–89 publications per year) as hot study periods. Notably, the citation trends show a more significant growth in 2018–2019 (169–593 citations per year) and 2020–2021 (1,472–2,798 citations per year), suggesting that the application of SC-EVs in the field of DM is receiving numerous attentions in general.

The global country distribution network of publications was visualized with VOSviewer, and the association strength method was used for normalization. The threshold for the minimum number of documents from a country was set at two, and a total of 23 countries met the threshold (Figure 2B). China (122 publications) was the most productive country, followed by the USA (50 publications) and Italy (16 publications). Subsequently, the density map was used to display all publishing centers more visually (Figure 2C). In terms of total citations, the top three countries were China (2,759 citations), the USA (1,167 citations), and Italy (366 citations). The countries with the highest total link strength were as follows: China (7,908), the USA (5,939), and Italy (3, 11), indicating that China and the USA have the predominant influence in this field. The top 10 countries with the most contributions are listed in Table 1. In addition, according to the year of concentration of country publications (Figure 2D), Mexico, the Netherlands, and New Zealand had publications mainly concentrated in 2017; The USA, Italy, and Japan had publications mainly concentrated in 2019; articles from China, Egypt, and India were mainly published after 2020.

**Table 1:** The top 10 countries that contributed publications on stem cell-derived extracellular vesicles (SC-EVs) for diabetes.

**Country**	**Publications**	**Citations**	**Average citation rate**
Peoples R China	122	2,759	22.61
Usa	50	1,167	23.34
Italy	16	366	22.88
Japan	14	219	15.64
India	11	167	15.18
Iran	10	299	29.90
South Korea	8	166	20.75
Egypt	8	127	15.88
Spain	7	159	22.71
Canada	6	42	7.00

### Institutional Distribution Analysis

The top five institutions with the most publications are located in Italy and China. Turin University (eight publications) was the most productive institution, followed by the Central South University (seven publications), Shanghai Jiaotong University (seven publications), Tianjin Medical University (seven publications), and Sun Yat Sen University (seven publications). According to citations, Shanghai Jiaotong University (306 citations) has the highest citations, followed by Central South University (242 citations) and Shangdong University (239 citations). The top 20 institutions with the most publications are listed in Table 2.

**Table 2:** The top 20 institutions with the most publications in the field of SC-EVs for diabetes.

			**Average**
			**citation**
**Institutions**	**Publications**	**Citations**	**rate**
Turin University	8	181	22.63
Central South University	7	41	5.86
Shanghai Jiaotong University	7	306	43.71
Sun Yat Sen University	7	117	16.71
Tianjin Medical University	7	114	16.29
Cairo University	6	126	21.00
Tongji University	5	173	34.60
Zhejiang University	5	205	41.00
Southern Medical University	5	74	14.80
Indian Inst Technol Kanpur	5	71	14.20
Harbin Medical University	5	69	13.80
Nantong University	5	48	9.60
Jinan University	5	43	8.60
Maryland University	4	70	17.50
Harvard Medical School	4	63	15.75
Peking University	4	36	9.00
Fourth Military Medical	4	28	7.00
University
Huazhong University of	4	14	3.50
Science and Technology
Cent S University	4	242	60.50
Shangdong University	4	239	59.75

**Figure 3:**
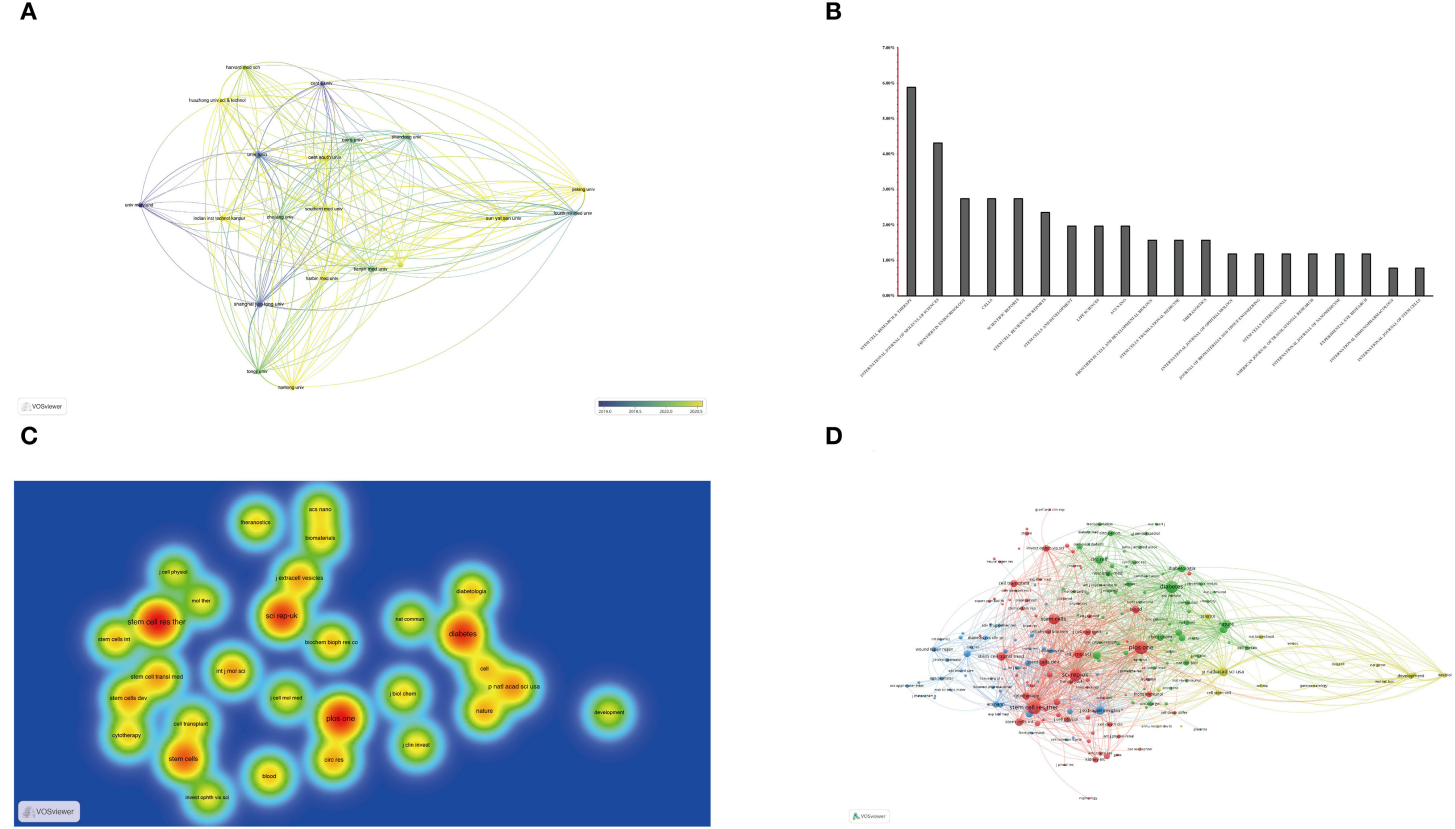
Articles on SC-EVs for diabetes published in different institutions and journals. **(A)** Collaboration between institutions based on VOSviewer. **(B)** The top 20 journals produced the largest number of articles. **(C)** Density map of article reference distribution based on VOSviewer. **(D)** Network visualization of article reference distribution based on VOSviewer.

Then, the close and complex collaborative relationships between the different institutions were analyzed with VOSviewer. The threshold for the minimum number of documents of an organization was set at four, and the top 20 institutions met the threshold and were presented in a network map by the year of concentration of institutional publications (Figure 3A). The result indicated that Turin University, Shanghai Jiaotong University, and Cent S University had publications mainly concentrated in 2019; articles from Sun Yat Sen University and Tianjin Medical University were mainly published after 2020.

### Journal Distribution Analysis

Journal distribution analysis helps to understand the hot journals in the field of SC-EVs for DM. The journal Stem Cell Research and Therapy (IF = 6.832, 2020) with 15 publications, and the journal International Journal of Molecular Sciences (IF = 5.924, 2020) with 15 publications, published the most studies. The journals Frontiers in Endocrinology (IF = 5.555, 2020), Cells (IF = 6.6, 2020), and Scientific Reports (IF = 4.35, 2020) had seven publications each. The top 20 journals with the most publications and the impact index of the top 10 journals with the largest number of articles are presented in Figure 3B; Table 3, respectively.

**Table 3:** Impact index of the top 10 journals with the largest number of articles.

		**Eigen**
	**Impact**	**factor**
**Journals**	**factor**	**Score**	**H-index**
Stem Cell Research & Therapy	6.832	76	76
International Journal of	4.556	98	162
Molecular Sciences
Frontiers In Endocrinology	5.555	No records	68
Cells	6.6	No records	22
Scientific Reports	3.998	100	213
Stem Cell Reviews and Reports	5.739	73	73
Stem Cells and Development	3.272	91	114
Life Sciences	5.037	89	164
Acs Nano	15.881	100	382
Frontiers in Cell and	6.684	No records	53
Developmental Biology

Then, reference co-citation analysis was performed to understand the close association between the referenced journals. The threshold for the minimum number of citations from a source was set at 20, and 210 journals met the threshold. The top 30 referenced journals were visualized in a density map, and all referenced journals were presented in a network visualization (Figures 3C,D). Stem Cell Research and Therapy, Scientific Reports, Diabetes (IF = 9.461, 2020), Plos One (IF = 3.24, 2020), and SCs (IF = 6.277, 2020) were the most thermal publication center, and they were most closely associated with other journals.

### Author Distribution Analysis

**Figure 4:**
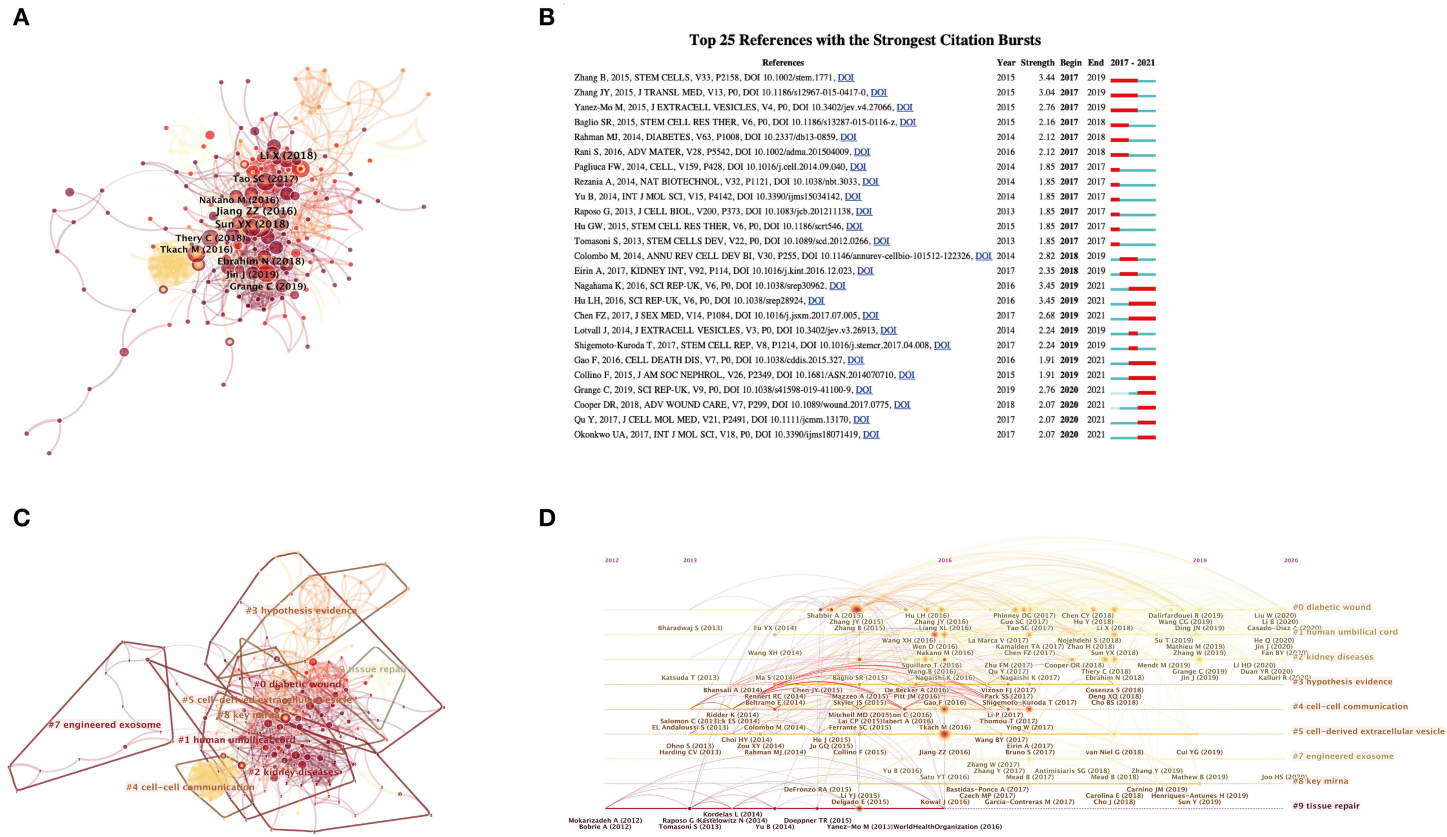
Mapping of references in studies on SC-EVs for diabetes. **(A)** A simplified co-citation network of references on SC-EVs for diabetes based on CiteSpace, and burst references are represented by red nodes. **(B)** The top 25 references with the strongest citation bursts based on CiteSpace. **(C)** Title clustering analysis of the co-citation network based on CiteSpace. **(D)** Timeline visualization of references from 2012 to 2020.

The most productive authors with the highest number of publications and citations from 2017 to 2021 are listed in Table 4. ASHOK KUMAR, from the Indian Institute of Technology Kanpur, India ranked first (five publications), and other authors from Italy, China, and Egypt followed with four publications each. The number of article citations is an important indicator of an author's influence. The top 10 authors with the most citations were all from China. QING LI, from the Chinese Academy of Sciences, published only two articles, the number of citations reached 307 in total, suggesting that he has drawn tremendous achievements and attention in the field of SC-EVs for DM research. Then, we used CiteSpace to analyze and visualize the co-citation network of the top 10 references of the shortlisted publications (Figure 4A). The article published by Sun et al. in ACS NANO in 2018 (doi: 10.1021/acsnano.7b07643) was a hub node in the co-citation network, followed by the article published by Li et al. in Experimental and Molecular Medicine in 2018 (doi: 10.1038/s12276-018-0058-5). To better understand the scientific frontiers of the field on SC-EVs for DM, we analyzed the references using the burst detection function (the minimum duration threshold was set as one) in CiteSpace. The top 25 references with the strongest citation bursts are presented in Figure 4B, and none of the articles had sudden changes in the number of citations in the last 3 years.

**Table 4:** The top authors in the field of SC-EVs for diabetes ranked by publication and citation numbers.

	**Author**	**Country**	**Affiliation**	**Publications**	**Citations**
Top publications (n ≥ 4)	Ashok Kumar	India	Indian Institute of Technology Kanpur	5	71
	Giovanni Camussi	Italy	University of Turin	4	99
	Fang Liu	China	Shanghai Jiao Tong University Affiliated Sixth People's Hospital	4	92
	Dina Sabry	Egypt	Cairo University	4	86
	Anamika Singh	India	Indian Institute of Technology Kanpur	4	70
	Chiara Gai	Italy	University of Turin	4	63
	Wei Wang	China	Central South University	4	43
	Xiao Lin	China	Central South University	4	25
	Yi Wang	China	Central South University	4	25
Top Citations (n ≥ 300)	Qing Li	China	Chinese Academy of Sciences	2	307
	Weiyang Gao	China	Second Affiliated Hospital and Yuying Children's Hospital of Wenzhou Medical University	2	303
	Bo lei	China	Xi'an Jiaotong University	2	303
	Cai lin	China	First Affiliated Hospital of Wenzhou Medical University	2	303
	Cong Mao	China	Second Affiliated Hospital and Yuying Children's Hospital of Wenzhou Medical University	2	303
	Chenggui Wang	China	Second Affiliated Hospital and Yuying Children's Hospital of Wenzhou Medical University	2	303
	Min Wang	China	Xi'an Jiaotong University	2	303
	Tianzhen Xu	China	Second Affiliated Hospital and Yuying Children's Hospital of Wenzhou Medical University	2	303
	Xingxing Zhang	China	First Affiliated Hospital of Wenzhou Medical University	2	303
	Ying Wang	China	Chinese Academy of Sciences	3	301

A title cluster analysis was implemented to generalize the references in the co-citation network to understand the frontier directions. The references in the co-citation network were divided into nine different clusters, including diabetic wound, human umbilical cord, kidney diseases, hypothesis evidence, cell–cell communication, cell-derived EV, engineered exosome, key miRNA, and tissue repair (Figure 4C). Based on the clustering display of the timeline, it can be found that the early publications focus on tissue repair and intercellular communication, and the publications in the last 5 years were mainly based on diabetic wound, kidney diseases, and engineered exosome. The top 10 references with the most total citations in the field of SC-EVs for diabetes are listed in Table 5.

**Table 5:** The top 10 references with the most citations in the field of SC-EVs for diabetes.

	**Corresponding**			**Publication**	**Total**	**Level of**
**Title**	**author**	**Journal**	**IF**	**year**	**citations**	**evidence**
Immunoregulatory Mechanisms of Mesenchymal Stem and Stromal Cells in Inflammatory Diseases	Ying Wang	NATURE REVIEWS NEPHROLOGY	28.314	2018	281	Review
Exosomes From Adipose-Derived Stem Cells (SCs) Attenuate Adipose Inflammation and Obesity Through Polarizing M2 Macrophages and Beiging in White Adipose Tissue	Qun Wang	DIABETES	9.461	2018	213	*In vitro*, animal
Engineering Bioactive Self-Healing Antibacterial Exosomes Hydrogel for Promoting Chronic Diabetic Wound Healing and Complete Skin Regeneration	Cong Mao	THERANOSTICS	11.556	2019	198	*In vitro*, animal
Chitosan Wound Dressings Incorporating Exosomes Derived from MicroRNA-126-Overexpressing Synovium Mesenchymal SCs Provide Sustained Release of Exosomes and Heal Full-Thickness Skin Defects in a Diabetic Rat Model	ChangQing Zhang	STEM CELLS TRANSLATIONAL MEDICINE	6.94	2017	147	*In vitro*, animal
Exosomal DMBT1 from human urine-derived SCs facilitates diabetic wound repair by promoting angiogenesis	Hui Xie	THERANOSTICS	11.556	2018	135	*In vitro*, animal
MSC-derived Extracellular Vesicles Attenuate Immune Responses in Two Autoimmune Murine Models: Type 1 Diabetes and Uveoretinitis	Ryang Hwa Lee	STEM CELL REPORTS	7.765	2017	131	*In vitro*, animal
Human Mesenchymal SC-Derived Exosomes Alleviate Type 2 Diabetes Mellitus by Reversing Peripheral Insulin Resistance and Relieving beta-Cell Destruction	Hui Qian	ACS NANO	15.881	2018	127	*In vitro*, animal
Exosomes from adipose-derived SCs overexpressing Nrf2 accelerate cutaneous wound healing by promoting vascularization in a diabetic foot ulcer rat model	Maoquan Li	EXPERIMENTAL AND MOLECULAR MEDICINE	8.718	2018	124	*In vitro*, animal, human
Efficient Angiogenesis-Based Diabetic Wound Healing/Skin Reconstruction through Bioactive Antibacterial Adhesive Ultraviolet Shielding Nanodressing with Exosome Release	Bo Lei	ACS NANO	15.881	2019	105	*In vitro*, animal
GMSC-Derived Exosomes Combined with a Chitosan/Silk Hydrogel Sponge Accelerates Wound Healing in a Diabetic Rat Skin Defect Model	Ximin Guo	FRONTIERS IN PHYSIOLOGY	4.566	2017	92	*In vitro*, animal

### Keyword Co-occurrence Cluster Analysis

**Figure 5:**
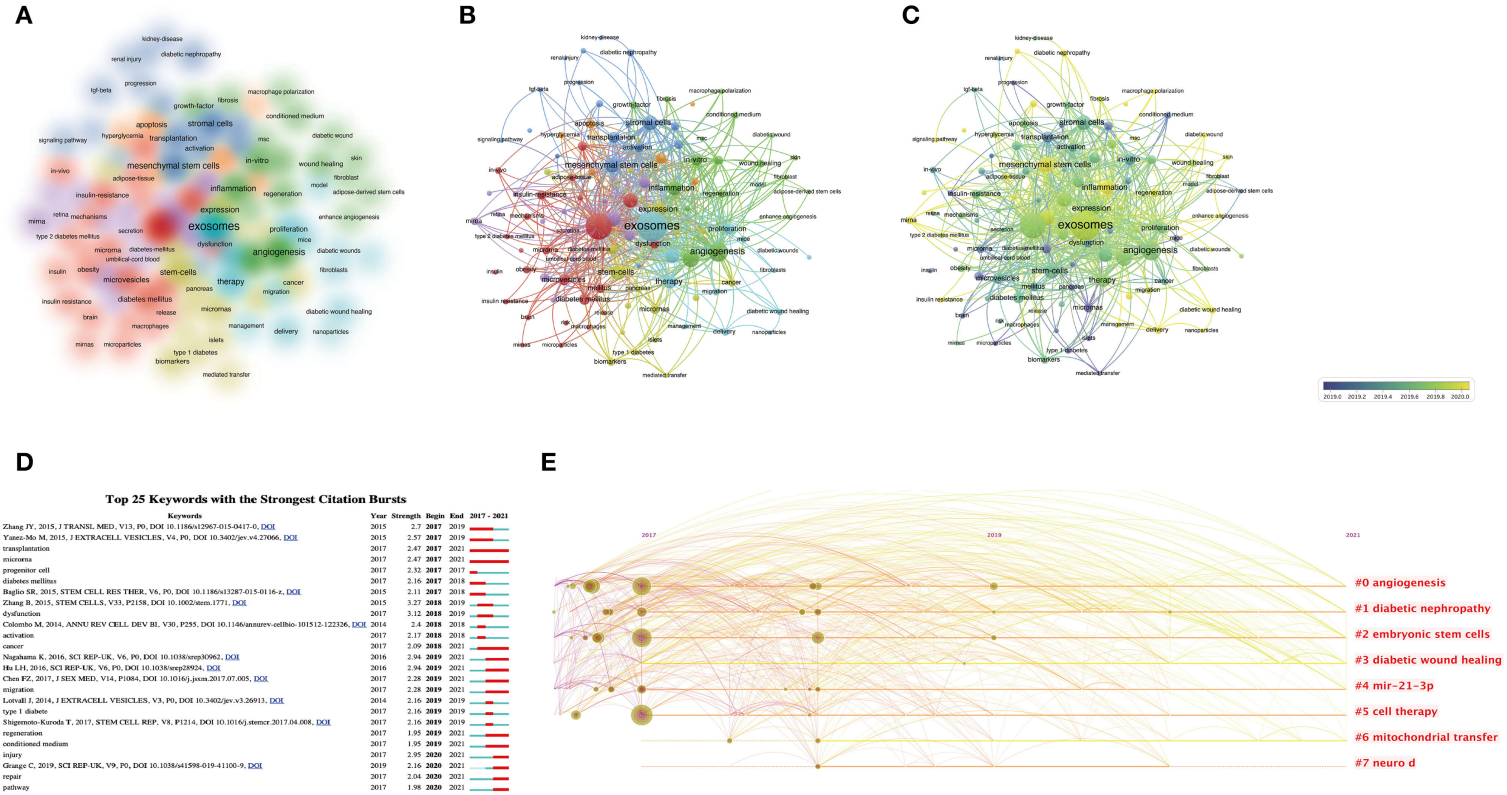
Keyword mapping in studies on SC-EVs for diabetes. **(A)** Clustering analysis of key words based on VOSviewer. **(B)** Network visualization of keywords based on VOSviewer. **(C)** Chronological order of keywords based on VOSviewer. **(D)** The top 20 keywords with the strongest citation bursts based on CiteSpace. **(E)** Keyword timeline visualization from 2017 to 2021.

Keywords represent the central topic of a publication, and keyword co-occurrence analysis helps to systematically understand the hot topics and current progress of SC-EVs for diabetes and their intrinsic connections. VOSviewer was used to analyze keywords, and the threshold was set at a minimum of five occurrence of a keyword in the titles and abstracts of the included publications. A total of 112 keywords were identified and were mainly divided into seven different clusters: EVs, exosomes, angiogenesis, SCs, mesenchymal SCs, diabetes, and oxidative stress (Figures 5A,B). In the overlay visualization, keywords were shown in different colors with their annual distribution according to the average year of publication (Figure 5C). For example, “microvesicles,” “conditioned medium,” and “microRNAs” appeared mainly in early 2019, while the keywords “inflammation,” “exosome,” and “angiogenesis” have emerged in recent years. The keywords that have emerged in recent years bode well for the growing popularity of these areas, which may become hot in the future. At the same time, co-occurrence cluster analysis was also performed in CiteSpace. The top 25 keywords with the strongest citation bursts (the minimum duration threshold was set as one) are presented in Figure 5D, and none of the keywords had sudden changes in the number of citations in the last 3 years. Based on the timeline clustering display, it can be found that angiogenesis, diabetic nephropathy, diabetic wound healing, embryonic SCs, miRNA, cell therapy, and mitochondrial transfer have been the hot topics of research in recent years and show the direction of future research.

## Discussion

Diabetes mellitus is a metabolic disease caused by islet dysfunction or insulin action disorder, and its incidence has increased steeply in the last decade (14). Long-term hyperglycemia causes complications in various organs such as kidneys, nerves, and blood vessels. It has the characteristics of slow onset, long duration, and prolonged disease progression, which eventually leads to the loss of self-care ability and even death of patients. At present, clinical control of diabetes is mainly through medication, diet control, and insulin injection therapy, and a combination with symptomatic treatment to control diabetic complications, but the effect is still unsatisfactory (2). Therefore, there is an urgent need for new therapeutic interventions for DM and its complications.

Published research in the field of diabetes continues to increase year by year, and the intervention and management of diabetes have attracted widespread attention (15). In recent years, SCs are hotspots of regenerative medicine research and have broad application prospects in the treatment of various complex diseases. The continuous breakthrough of SC technology is expected to change the existing clinical treatment model and become a new biological therapy following traditional treatments, such as drugs and surgery (16). However, the potential tumorigenic risk and the difficulties of storage and transport of SCs have led to slow advancement in the clinical translation of cell therapy. EVs are nanosized particles secreted by cells, which contain a variety of biologically active substances, such as lipids, proteins, and RNAs, and play an important role in intercellular communication, cell survival and apoptosis, and the regulation of disease progression. Interestingly, numerous previous studies have shown that SC-EVs, especially exosomes, have repairing effects similar to SCs without the risk of tumor formation and can be stably preserved for transport, which has attracted a great deal of attention from researchers (2, 17). To explore the process and trend of SC-EVs for DM, this study conducted a bibliometric analysis of the publication on SC-EVs for DM from 2017 to 2021.

Bibliometrics is now widely used in various fields of global research, helping researchers to gain an intuitive and systematic understanding of a field and to identify significant scientific achievements and future research hotspots. Statistics on the number and citation frequency of SC-EVs used in DM publications show that the number of publications and the frequency of citations have increased significantly over the past 5 years, indicating that this field is a current research hotspot (Figure 2A). Notably, more than half of the top 20 journals in this field have an IF above five, which means that the topic of SC-EVs for DM has attracted a lot of attention (Figure 3B). Therefore, more in-depth studies will be conducted to explore the feasibility and mechanism of action of SC-EVs for the treatment of DM.

To better understand the national and regional contributions in this field, a national distribution analysis of publications was performed. China ranks first in the world in terms of total number of publications and citations, but the average citation rate in China (22.61) is lower than that of the USA (23.34), Iran (29.9), and Italy (23.34), which means that China still needs to improve the quality of publications (Table 1). A recent bibliometric study on diabetes showed that India, the USA, China, South Korea, and Brazil were the most productive countries (18). Among them, the average citation rate of the USA remains at the top, but China's development trend is strong and is expected to overtake the USA. The contribution of research institutions is an important part of the country's contribution in this field. For the total publication volume, 75% of the top 20 institutions are located in China, with Shanghai Jiaotong University having the highest number of citations (Table 2). In terms of temporal distribution (Figure 2D), China mainly focuses on the use of SC-EVs for the treatment of diabetes after 2020, which shows that China has attached great importance to the application of SC-EVs as a biological therapy for the treatment of DM and its complications in recent years.

In terms of author distribution (Table 4), ASHOK KUMAR (Indian Institute of Technology Kanpur) from India is the most productive and has a certain number of citations. GIOVANNI CAMUSSI (University of Turin) from Italy published the second highest number of papers, but has the highest number of citations in the top publishing authors. Notably, QING LI (Chinese Academy of Sciences) from China has published only two studies in this field, but the number of citations is as high as 307 citations, which is the highest average citation rate among all researchers. The article by Shi (doi: 10.1038/s41581-018-0023-5) had the largest number of citations in the co-citation network, and Ying Wang was the corresponding author (Table 5). However, Ying Wang and his team mainly reviewed the immunomodulatory mechanism of mesenchymal SCs and their therapeutic applications, and did not provide a detailed overview of the application of SC-EVs in the field of DM (19). The articles by Sun (doi: 10.1021/acsnano.7b07643) and Li (doi: 10.1038/s12276-018-0058-5) are hub nodes in the co-citation network (Figure 4A). Sun et al. (20) found that human umbilical cord mesenchymal SC-derived exosomes reversed peripheral insulin resistance and attenuated islet β-cell destruction in type 2 diabetes by improving glucose utilization in peripheral tissues, increasing glycogen storage in the liver, and reducing islet β-cell apoptosis. Li et al. (21) applied adipose SC-derived exosomes to diabetic foot ulcers and found that adipose SC-derived exosomes accelerate chronic wound healing by promoting wound vascularization, epithelialization, and reducing inflammation and oxidative stress. It is evident that SC-EVs, especially exosomes, have broad application prospects in the treatment of DM and its complications.

A title cluster analysis of the references and a keyword co-occurrence cluster analysis of the publications were performed to summarize the latest hot trend of SC-EVs for DM. For the cluster analysis of references and distribution in a timeline (Figure 4D), researchers were particularly interested in EVs for intercellular communication and organizational repair research in 2016, and then researchers seemed to focus more on the microRNA repair mechanism of EVs and the clinical transformation of engineered exosomes. For instance, Yan et al. (22) used milk-derived exosomes as a delivery medium for miR-31-5p, which could effectively promote the healing of diabetic wounds. In terms of the cluster analysis of publications and their distribution in a timeline (Figures 5B,E), it is not difficult to find that SC-derived exosomes can improve DM and its complications (especially diabetic wounds and diabetic nephropathy) through various mechanisms such as improving insulin resistance, promoting vascularization, and regulating inflammation, which is a current research hotspot.

This study extracted the relevant publications on SC-EVs for DM and its complications in the WoS database and fully analyzed the current hotspot trend, but there is still a certain limit. For example, we only analyze English language publications, resulting in possibly ignoring non-English quality documents. Therefore, subsequent collaboration with researchers from other countries should be initiated to achieve more in-depth and comprehensive analysis results.

## Conclusion

In summary, this study provides a comprehensive summary and analysis of the global research trends of SC-EVs used in DM and its complications. In the past 5 years, relevant high-quality publications in this field have increased significantly, and SC-EVs s have a good prospect for development in the treatment of DM and its complications.

## Data Availability Statement

The original contributions presented in the study are included in the article/Supplementary Material, further inquiries can be directed to the corresponding author/s.

## Author Contributions

JX and YY were responsible for the study design and administrative support. HQ and RG were responsible for data collection. JY were responsible for analyzing the data. JX and HQ were responsible for manuscript drafting. YZ revised this manuscript. All authors critically reviewed and approved the final manuscript.

## Funding

This work was funded by the National Natural Science Foundation of China (Grant No. 81971089).

## Conflict of Interest

The authors declare that the research was conducted in the absence of any commercial or financial relationships that could be construed as a potential conflict of interest.

## Publisher's Note

All claims expressed in this article are solely those of the authors and do not necessarily represent those of their affiliated organizations, or those of the publisher, the editors and the reviewers. Any product that may be evaluated in this article, or claim that may be made by its manufacturer, is not guaranteed or endorsed by the publisher.
