# Supplementary material for: A Bibliometric Analysis of the Hotspots Concerning Stem Cell Extracellular Vesicles for Diabetes in the Last 5 Years
Source: Front Public Health. 2022 Jun 2;10:868440. doi: 10.3389/fpubh.2022.868440 (PMC9201211; doi:10.3389/fpubh.2022.868440)
Supplement: Supplementary file 1 [file Image_1.pdf]

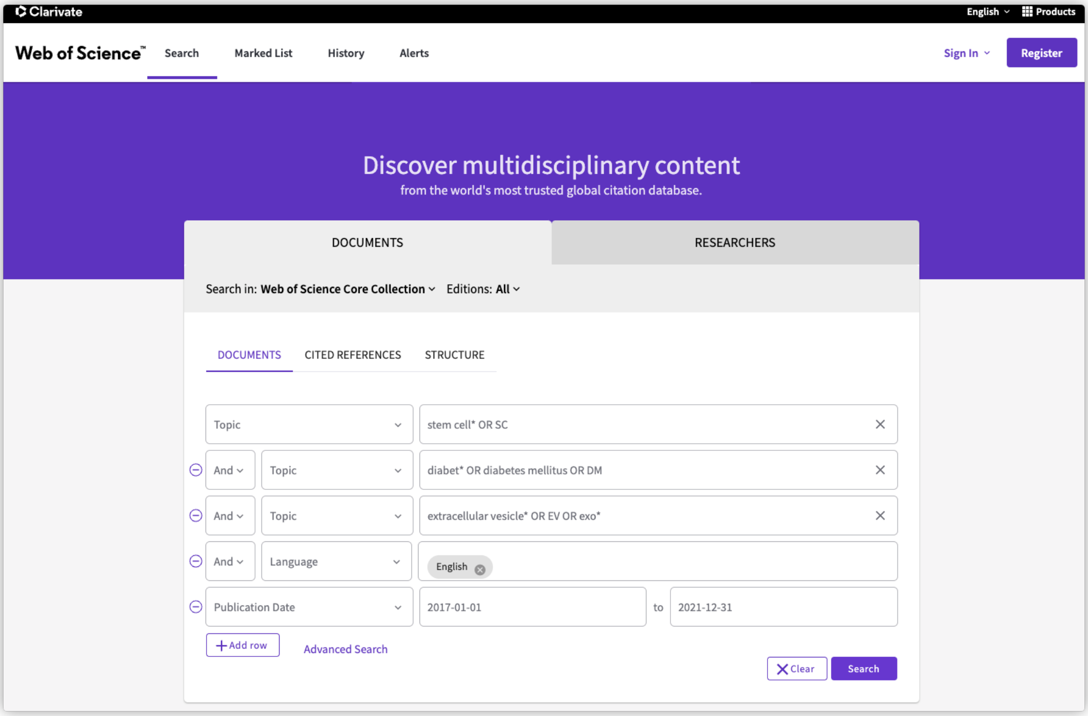

Figure 1: the screenshots of the search methodology in the Web of Science.
